# Free operant observing in humans: a translational approach to compulsive certainty seeking

**DOI:** 10.1177/1747021817737727

**Published:** 2018-01-01

**Authors:** Sharon Morein-Zamir, Sonia Shahper, Naomi A Fineberg, Verena Eisele, Dawn M Eagle, Gonzalo Urcelay, Trevor W Robbins

**Affiliations:** 1 Psychology Department, Anglia Ruskin University, Cambridge, UK; 2 Behavioural and Clinical Neuroscience Institute, University of Cambridge, Cambridge, UK; 3 Department of Psychology, University of Cambridge, Cambridge, UK; 4 Highly Specialized Obsessive Compulsive and Related Disorders Service, Hertfordshire Partnership University NHS Foundation Trust, Welwyn Garden City, UK; 5 Hertfordshire Partnership University NHS Foundation Trust, Welwyn Garden City, UK; 6 Postgraduate Medical School, University of Hertfordshire, Hatfield, UK; 7 Department of Neuroscience, Psychology and Behaviour, University of Leicester, Leicester, UK

**Keywords:** Checking, intolerance of uncertainty, obsessive compulsive disorder, observing

## Abstract

Excessive checking is reported in non-clinical populations and is a pervasive symptom in obsessive compulsive disorder (OCD). We implemented a free-operant task in humans, previously used in rats, wherein participants can “check” to reduce uncertainty. Participants can press an observing key to ascertain which of two main keys will, if pressed, currently lead to rewards. Over a series of experiments, we found that punishment robustly increased observing in non-clinical participants and that observing persisted long after punishment was removed. Moreover, participants appeared insensitive to the initial costs of checking, and a threefold increase in the effort required to observe served to deter participants only to a limited degree. We also assessed observing in OCD patients with no known comorbidities. The patients observed more than control participants and were abnormally insensitive to the introduction of punishment. These findings support the translational value of the task, with similar behaviours in humans and rodents. This paradigm may serve as a unifying platform, promoting interaction between different approaches to analyse adaptive and maladaptive certainty seeking behaviours. Specifically, we demonstrate how seemingly disparate theoretical and empirical approaches can be reconciled synergistically to promote a combined behavioural and cognitive account of certainty seeking.

Seeking greater certainty in the face of unpredictability or doubt is commonplace and often an effective approach in uncertain or ambiguous situations. Repeated checking characterises 15% of the normal population (Stein, Forde, Anderson, & Walker, 1997). When excessive, as in compulsive checking, it can lead to considerable distress and impaired everyday life functioning. Obsessive compulsive disorder (OCD) characterised by obsessions and compulsions (American Psychiatric Association [APA], 2013), has long been conceptualised as a disorder of doubt (Pitman, 1987; Rachman, 2002). Time-consuming checking is one of the most common symptoms with prevalence estimates ranging from 50% to 80% (Antony, Downie, & Swinson, 1998; Henderson & Pollard, 1988).

Multiple determinants likely play a role in development, escalation, and maintenance of excessive checking. As such, various psychological, behavioural, and cognitive neuroscience accounts have been proposed. Checking may arise in response to obsessions, intrusive thoughts, and fears focusing on impending threat and harm to one’s self or others (APA, 2013). Compulsive checking may also be associated with impairments in the ability to behave in a goal-directed manner, with increased reliance on habitual actions (Gillan & Robbins, 2014). Behavioural inflexibility and inhibitory difficulties may also contribute to compulsive checking (Chamberlain, Blackwell, Fineberg, Robbins, & Sahakian, 2005; Snyder, Kaiser, Warren, & Heller, 2015), as may impairments in terminating security-related behaviours (Szechtman & Woody, 2004). Compulsivity more broadly, is believed to involve the dysfunction of parallel, partly segregated, cortico-striato-thalamo-cortical circuits (van den Heuvel et al., 2016), with pharmacological and genetic evidence pointing to multiple neurochemical system alterations, including in dopamine, serotonin, and glutamate (Albelda & Joel, 2012; Zai, Brandl, Müller, Richter, & Kennedy, 2014). One key challenge is to integrate findings from disparate approaches and different levels of analyses.

A potential useful construct in this regard is certainty seeking. Certainty seeking, incorporating checking, and reassurance seeking behaviours are found not only in OCD but also in anxiety disorders, and in other obsessive compulsive and related disorders such as body dysmorphic disorder (Carleton et al., 2012; Coleman, Pieterefesa, Holaway, Coles, & Heimberg, 2011). Active certainty seeking, involving reliance on environmental cues, may be elicited as a coping strategy to alleviate distress due to perceived uncertainty, threat, and excessive doubting. Ambiguity or uncertainty can be aversive to humans and other primates (Hayden, Heilbronner, & Platt, 2010; Hsu, Bhatt, Adolphs, Tranel, & Camerer, 2005). Intolerance of uncertainty (IU) is a related trans-diagnostic concept, initially developed in relation to anxiety but increasingly posited as central to checking, washing, and other OCD symptoms (Birrell, Meares, Wilkinson, & Freeston, 2011; Lind & Boschen, 2009; Sarawgi, Oglesby, & Cougle, 2013). IU refers to experiencing doubt as aversive, whereby high IU individuals find even moderate uncertainty stressful or upsetting (Birrell et al., 2011). There is an association between IU and checking compulsions and with OCD patients also reporting high levels (Holaway, Heimberg, & Coles, 2006; Tolin, Abramowitz, Brigidi, & Foa, 2003). While checking, doubt, and IU seem inherently linked, the relation between them appears complex. For example, not only can doubt and IU contribute to checking, but checking may paradoxically promote doubt, reduced cognitive confidence, and even increased IU (Coleman et al., 2011; Lambrecq et al., 2014; van den Hout & Kindt, 2003).

Research measuring checking has assessed healthy volunteers and patients using introspection and retrospective self-report, and with memory and decision-making tasks which examine the extent to which participants re-check task-relevant information (Harkin & Kessler, 2009; Kim et al., 2012; Rotge et al., 2008; van den Hout & Kindt, 2003). An advantage of behavioural tasks is in allowing the examination of neural correlates of checking, with a recent study reporting extensive cortical activations in OCD and healthy volunteers (Rotge et al., 2015). Checking poses a challenge inasmuch as participants have to check *something* (e.g., whether two stimuli are the same, or whether one locked the door). Thus, such tasks have had to address the role of complex cognitive processes in the checking behaviours assessed, such as visuo-spatial processing, memory for actions, or working memory.

These lines of inquiry have advanced largely independently of the burgeoning animal research (Albelda & Joel, 2012; Eagle et al., 2014; Szechtman et al., 2016). Checking has been explored in rodent models of compulsivity where genetics, lesion, and pharmacological challenges can be used in ways not possible in humans. Following chronic quinpirole treatment (a dopamine D2/3-receptor agonist), rats display many repetitive features characteristic of human compulsive checking in an open-field environment (Szechtman, Sulis, & Eilam, 1998). Recently, an operant task to examine cognitive processes involved in checking has been developed and validated in rodents (Eagle et al., 2014). Based on earlier observing tasks (Shahan, 2002; Wyckoff, 1952), rats could press for food pellets on either of two levers, but only one was active at a given time. Additionally, rats could press an “observing” lever to obtain information about which of the two levers was presently active. Naturally high checking rats used the observing cue to locate the active lever thereby reducing task uncertainty and improving their performance. Chronic administration of quinpirole selectively increased functional and non-functional observing, analogous to compulsive checking, without influencing the main lever responses. Moreover, the excessive observing was reduced following sulpiride administration (a dopamine D2/3-receptor antagonist) implicating dopamine D2/3 receptor activation in generating excessive checking.

This study presents a line of experiments translating the Observing Response Task (ORT) to humans to assess its utility as a unifying behavioural model of checking. As in the rodent task, this is a *free operant* task where participants can press left and right buttons to earn rewards. The buttons alternate between reinforcement and extinction schedules, so that only one button is active and leads to rewards at a given time. A third, observing button activates a cue, allowing participants to ascertain which of the two main buttons is active. Participants respond to gain greater certainty in an open-ended yet still controlled situation, amenable to behavioural analysis (Szechtman & Woody, 2004). In Experiment 1, we assessed whether factors, likely to increase a sense of uncertainty or threat, influence observing in humans in the same way as in rodents and whether a similar role for individual differences can be detected (Eagle et al., 2014). In Experiment 2, we explored what role observing costs may play in circumstances characterised by threat uncertainty where avoidance, so characteristic of OCD, may contribute to certainty seeking. In Experiment 3, we examined whether individuals can adapt their ongoing checking levels. We then turned to assess whether or not OCD patients indeed observe more, as would be expected.

## Experiment 1

Based on key parametric task manipulations in the rodent ORT, Experiment 1 examined the consequences of uncertainty by altering various task demands (Eagle et al., 2014). Reinforcement uncertainty was assessed by changing the requirements to obtain reinforcement compared with baseline, with a greater number of active key presses needed to earn rewards. Uncertainty was also assessed by increasing the unpredictability in active key location, making the observing schedule less predictable. Finally, as noted above, as compulsions are often avoidant in OCD we included a condition where pressing the inactive key would lead to punishment. We anticipated that all three manipulations would lead to increased observing levels (Eagle et al., 2014; Shahan, 2002). Following the individual variation in observing behaviour noted in rodents, we confirmed this in the human version. Consequently, individuals were assigned to high and low observing groups using a median split, to investigate this further.

### Method

#### Participants

Seventy nine participants (53 female) with a mean age of 23.69 years (standard deviation [SD] = 2.94) participated and were compensated at a rate £8 per hour. All had normal or corrected-to normal vision and hearing, no psychiatric conditions and were free from psychotropic medication. The four groups (see below) did not differ in age, *F*(3, 75) = 0.99, *p* = .40, or gender distribution, χ^2^(3) = 1.41, *p* = .70.

#### Stimuli and apparatus

**Figure 1.**
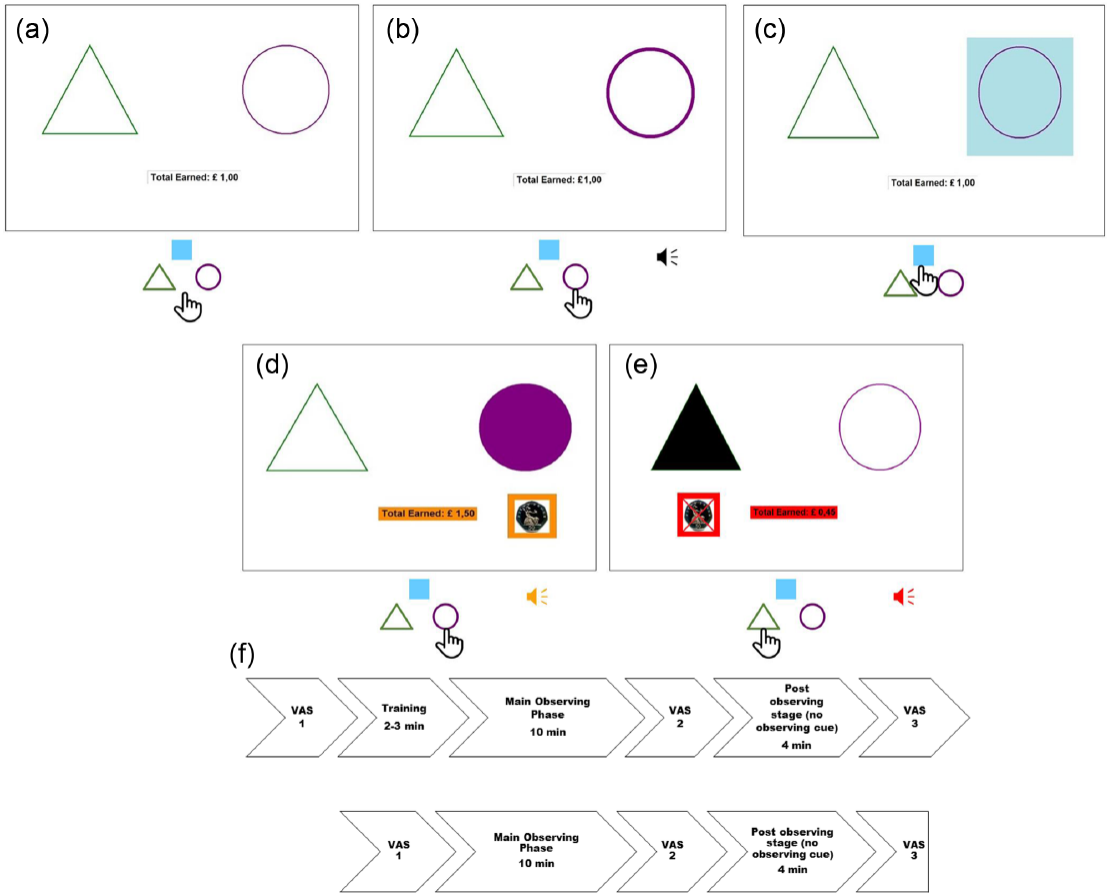
Schematic figures of the free operant observing task and procedure. At any given time, only one of the sides (and hence shapes) is active, thereby yielding rewards, while the other side/stimuli is inactive. (a) Participants can press either of two keys to earn rewards. (b) Key presses lead to the shape outline on the active side becoming briefly thicker and are accompanied by a click. (c) Participants may press the centre blue observing key to check which side is currently active. Observing results in a light blue square appearing (1.5 s) behind the shape on the active side. (d) Rewards are conveyed by a filled symbol on the active side, points and an uplifting noise. In the punishment condition, pressing on the inactive side yields punishment (see text for details). (e) Punishments are conveyed by a symbol filled in black on the inactive side, loss of points and an aversive noise. The procedure involves two successive sessions comprising of a main observing phase followed by briefer post observing stage where the observing cue is extinguished. (f) Participants also rate their current level of anxiety on a visual analog scale (VAS) throughout the task.

The main visual display contained the outline of a green triangle (sides 7.1°) and purple circle (diameter 6.4°) to either side of the screen (Figure 1a). Shape location was counterbalanced within each group. The text “Total Earned:” and the appropriate amount appeared in a rectangle (7° by 1°) centrally below. Pressing the “m” and “z” keys (labelled with the corresponding coloured shape) led to a brief click accompanied by a thicker outline (Figure 1b). A “t” keypress caused a light-blue square (8.3° by 8.3°) to appear behind the active shape in observing phases (Figure 1c). Rewards were denoted by a 50 pence coin below the rewarded side within an orange frame, accompanied by an uplifting sound (duration 400 ms). Additionally, the earnings box turned orange and the active side shape became filled (Figure 1d). Punishment was a crossed-out coin in a red frame, accompanied by an aversive shrill noise (250 mc). The reinforcer utilised multiple modalities to ensure saliency and elicit motivation throughout the task. Furthermore, the earnings box turned red and the inactive side shape filled with black (Figure 1e). Visual analogue scale (VAS) ratings were performed with a horizontal black line (coded from 1 to 30) against a light-grey background. The experiment, programmed using Visual Studio, was conducted on a 15.6″ laptop with connected mouse, running Windows 7.

#### Procedure and design

Participants were seated at a comfortable viewing distance and tested individually with the experimenter present. They were told to earn as much as possible, but that at any given time only one side will be active. Instructions indicated that the active side will alternate over time and pressing the key on the active side will result in rewards while the other side will not. Instructions noted participants would receive a proportion of their earnings (in practice this was always £5). Training lasted 2-3 min, with participants first presented with one shape (counterbalanced across participants) and allowed to earn several rewards. Then, for 1 min, both sides were presented with the observing cue present and shifting as the active side changed. Finally, the cue was removed and participants informed they could press the observing key to produce it, and were asked to do this at least once. At the onset of the observing stage, which lasted 10 min, instructions noted that pressing the observing key would produce the cue but at a small cost. After a self-terminating break, participants received instructions and practised the post-observing stage (4 min) where the cue was not available. Analyses of this stage are reported for all experiments in supplemental materials. Following a brief break, participants completed another session with baseline parameters. Throughout, participants rated how they were “feeling right now” on a VAS ranging from very calm to very anxious three times (see Figure 1f).

Observing responses yielded the cue for 1.5 s. Rewards and punishments were £0.50 and observing responses cost £0.05. Response feedback was immediate with visual feedback of non-reward responses lasting 250 ms and of rewards and punishments lasting 400 ms. No limitations were placed on responses, with the exception that participants could not press more than 4 times a second whereupon an error signal sounded and they were asked to refrain from going too fast. Sample size was selected on the basis of previous observing and free-operant research. Participants provided written informed consent, and the study was approved by the University Ethics Board.

Baseline was compared with three conditions in a between-subjects design. In baseline, each side when active, yielded rewards on a variable ratio (VR) of 14 whereby rewards were available on average every 14 presses (range 6-22). The active side switched according to a variable time (VT) schedule of 16 s whereby on average the active side shifted every 16 s (range 4-28). The increased effort (reinforcement uncertainty) condition was identical with the exception that it used a VR30 schedule (range 4-56). The increased schedule unpredictability condition differed from baseline in that the active side alternated more frequently (VT10, range 1-19 s). Punishment differed from baseline in that rather than there being no consequences when participants pressed the inactive button, it now yielded punishment on a VR14 (6-22) schedule. Primary outcome indices were observing presses, main button presses (MBP), and anxiety ratings, with the first two reported in rates per minute as in the rodent version. Secondary measures consisted of reward rate, total earnings, and where relevant punishment rate. Inspection of performance revealed considerable variation in observing levels similar to rodent findings (Eagle et al., 2014). In accordance with the approach previously adopted, a median split for each group was performed resulting in high and low observing styles. Thus, the design included four levels of condition: baseline, increased effort, increased unpredictability and punishment, crossed with low and high observers. MBP included side (active vs inactive) as an additional factor. After the task, participants completed several questionnaires with Latin square counterbalancing. The Beck Depression Inventory (BDI; Beck, Ward, Mendelson, Mock, & Erbaugh, 1961), Obsessive Compulsive Inventory (OCI-R; Foa et al., 2002), State/Trait Anxiety Inventory (STAI; Spielberger, Gorsuch, Lushene, Vagg, & Jacobs, 1983), and IU (Buhr & Dugas, 2002) were included. The Padua, Metacognitive Questionnaire, and Multidimensional Perfectionism Scale were administered but not analysed (Burns, Keortge, Formea, & Sternberger, 1996; Frost, Marten, Lahart, & Rosenblate, 1990; Wells & Cartwright-Hatton, 2004).

### Results

#### Observing stage

##### Observing

**Figure 2.**
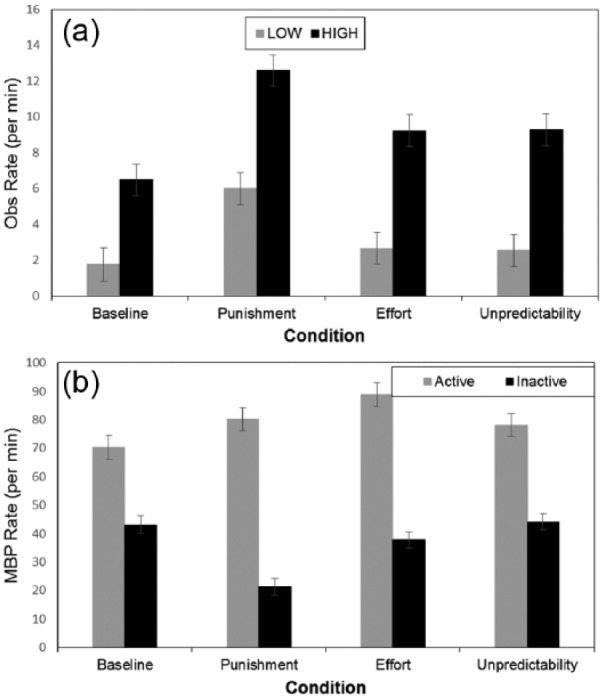
(a) Mean observing rate for high and low observers. (b) Mean button press for active and inactive sides in Experiment 1. OBS: observing; MBP: mean button press Error bars depict the standard error of the mean.

A 4 x 2 two-way analysis of variance (ANOVA) was conducted with condition and observing style (Figure 2). Median observing rates per minute were 4.15 for baseline, 4.74 for increased effort, 5.15 for increased unpredictability, and 9.66 for the punishment condition. There was an effect of condition, *F*(3, 71) = 5.01, *p* = .003, η_p_^2^ = 0.17, stemming from higher observing rates with punishment, *F*(1, 71) = 29.96, *p* < .001. As expected observing style was significant, *F*(1, 71) = 95.24, *p* < .001. Planned comparisons indicated higher observing in high observers compared to baseline under increased effort, *F*(1, 71) = 4.83, *p* = .03, and under increased unpredictability, *F*(1, 71) = 5.03, *p* = .03. In low observers, higher observing rates were only found with punishment compared to baseline, *F*(1, 71) = 10.92, *p* = .001.

##### MBP

A 4 x 2 x 2 ANOVA with condition, side (active vs. inactive), and observing style indicated effects of condition, *F*(3, 71) = 5.16, *p* = .003, η_p_^2^ = 0.18; side, *F*(1, 71) = 267.10, *p* < 0.001, η_p_^2^ = 0.79; and the interaction between them, *F*(3, 71) = 7.90, *p* = .0001, η_p_^2^ = 0.25 (Figure 2b). Follow-up comparisons indicated greater active MBP rates in the effortful condition compared to baseline, *F*(1, 71) = 9.89, *p* = .002, but no difference in inactive MBP, *F*(1, 71) = 1.67, *p* = .20. Additionally, under punishment compared to baseline there were decreased inactive MBP, *F*(1, 71) = 27.48, *p* < .001, but no difference in active MBP, *F*(1, 71) = 2.77, *p* = .10. There was no effect for observing style, *F*(1, 71) = 1.48, *p* = .23, but an interaction between side and observing style, *F*(1, 71) = 68.58, *p* < .001, η_p_^2^ = 0.49, stemming from a smaller difference between sides for low compared with high observers. Thus, high observers focused their efforts in the active side compared with low observers, indicating the utility of observing.

##### Rewards and punishments

An ANOVA on rewards with condition and observing style indicated effects for condition, *F*(3, 71) = 14.76, *p* < .001,η_p_^2^ = 0.38; observing style, *F*(1, 71) = 11.85, *p* < .001,η_p_^2^ = 0.14; and no interaction, *F*(3, 71) = .42, *p* = .74. Post-hoc analyses indicated lower reward rates under increased effort (*M* = 2.09, *SD* = 0.58), compared to baseline (*M* = 3.91, *SD* = 1.42), with no difference between the latter and increased unpredictability (*M* = 4.13, *SD* = 1.16) or punishment conditions (*M* = 3.86, *SD* = 1.31). This was expected given the reward schedule for increased effort. Additionally, high observers earned slightly more rewards (*M* = 3.91, *SD* = 1.43) than low observers (*M* = 3.06, *SD* = 1.26). In punishment, high observers received fewer punishments (*M* = 0.29, *SD* = 0.19) than low observers (*M* = 0.86, *SD* = 0.52).

#### Earnings

An ANOVA with condition and observing style indicated only a significant effect for condition, *F*(3, 71) = 17.54, *p* < .001,η_p_^2^ = 0.43. There was no significant observing style effect, *F*(1, 71) = 2.53, *p* = .116, and no interaction, *F*(3, 71) = .52, *p* = .67. Earnings were £7.52 (*SD* = 3.23) for increased effort, £17.67 (*SD* = 4.54) for the uncertain condition, £11.74 (*SD* = 5.92) for the punished condition and £17.58 (*SD* = 6.65) for baseline. *Post-hoc* Tukey’s tests indicated reduced earnings for both increased effort and punishment compared to baseline (*p*s < .001).

#### Self-reported anxiety

An ANOVA included condition, observing style as between-subjects factors and experiment stage as a within-subjects factor. Experiment stage was significant, *F*(2, 142) = 5.78, *p* = .004, η_p_^2^ = 0.07, and it interacted with condition, *F*(6, 142) = 4.49, *p* < .001, η_p_^2^ = 0.16. Simple interactions indicated a gradual increase in anxiety over time for baseline and punishment, *F*(2, 70) = 11.30, *p* < .001, with values increasing from *M* = 7.83, through *M* = 9.66 to *M* = 11.66. There was no change in the remaining conditions, *F*(2, 72) = .79, *p* = .456, with values being *M* = 7.65, *M* = 6.95 to *M* = 7.12, for first, second, and third measurements, respectively.

#### Follow-up session

The follow-up session was examined to ascertain whether participants would retain their observing strategy. We therefore report observing in the observing stage and self-reported anxiety across the whole session.

##### Observing

A two-way ANOVA indicated effects for condition, *F*(3, 71) = 4.59, *p* = .005, η_p_^2^ = 0.16, and observing style as derived from the first session, *F*(1, 71) = 46.16, *p* < .001, η_p_^2^ = 0.39, but no interaction, *F*(3, 71) = .231, *p* = .874. There were greater observing rates under punishment (*M* = 8.08, *SD* = 4.40) compared to baseline (*M* = 4.40, *SD* = 4.13). Observing did not differ from baseline (*p* > .5) in increased effort (*M* = 4.84, *SD* = 5.10) and greater unpredictability (*M* = 5.42, *SD* = 3.88). Those who observed more in the first session, continued to do so compared with previously low observers (*M* = 8.32 vs *M* = 2.97, respectively).

##### Self-reported anxiety

The ANOVA included condition, observing style and stage. Stage was significant, *F*(2, 142) = 4.17, *p* = .017, η_p_^2^ = 0.06, and it interacted with condition, *F*(6, 142) = 4.65, *p* = .0002, η_p_^2^ = 0.16. The interaction stemmed from decreased anxiety over time for the previous punishment, *F*(1, 71) = 28.12, *p* < .001, with no difference in the other conditions (*p* > .4).

#### Questionnaire data

Self-reported levels of depression, IU, OC symptoms, and anxiety and age did not correlate with task performance as assessed by observing, MBP, or earnings (see supplemental materials). Mean VAS anxiety correlated positively with STAI state, *r* = .44, *t*(77) = 4.28, *p* < .001; trait, *r* = .40, *t*(77) = 3.85, *p* < .001; and depression, *r* = 0.28, *t*(77) = 2.56, *p* = .013. We noted that all measures differed between the four groups. This was confirmed by a multivariate ANOVA, Wilks Lambda = 0.613, *F*(15, 196.40) = 2.53, *p* < .01. Depression, IU, and trait anxiety were significant (all *p*s < .05) with OC symptom severity showing the same trend (*p* < .07). *Post-hoc* Tukey’s tests revealed that the punishment condition led to reporting higher symptom levels compared to either baseline, greater uncertainty, or both.

#### Summary of results

Threat uncertainty (possibility of punishment) led to greater observing rates regardless of response style. In contrast, greater reinforcement uncertainty and increased unpredictability in active button location did not lead to robust increased observing, though there was some suggestive evidence of this in high observers. Individual differences were evident in observing style. Though high observers earned more rewards and avoided punishments, this was offset by the cost of observing, so that increased observing did not translate into greater earning. Observing was largely functional, and was associated with more active side responses and greater inactive side avoidance. Thus, greater observing was associated with more rewards overall, *r* = .37, *t*(76) = 3.50, *p* < .01, and under threat uncertainty, with reduced punishment rates, *r* = –.71, *t*(18) = –4.27, *p* < 0.01. Removal of the observing cue (see supplemental materials) revealed that participants had not learned the task contingencies and could not differentiate between the active and inactive sides. Participants in the punishment condition only now pressed less and earned fewer rewards. In the follow-up session observing remained largely stable within individuals with a strong association between the two sessions, *r* = .82, *t*(77) = 12.76, *p* < .001. Moreover, though informed of the removal of punishment, participants did not consequently reduce observing, despite the cost. Observing levels did not correlate with self-reported measures. Self-reported trait IU, anxiety, and depression were reported as higher when following punishment.

### Discussion

The results demonstrate the translational value of the ORT. Similar to rodent findings, humans could perform the task and observe at will (Eagle et al., 2014; Shahan, 2002). Participants appeared to observe to reduce uncertainty with greater observing associated with increased active side and reduced inactive side responding. As found in rats, humans also employed different strategies with some electing to observe more than others. This was not indicative of differences in overall motivation as MBP were similar, and more effective responding was offset by observing costs so that with the present outcome contingencies high observers did not earn more than low observers.

We employed two manipulations used in rodents to increase uncertainty. Previously in rats, greater reinforcement uncertainty resulted in increased observing with parameters similar to those employed here (Eagle et al., 2014). When active lever location was more unpredictable, only high checking quinpirole treated rats increased their observing rates. In humans, manipulations that increased reinforcement uncertainty (increased effort and unpredictability) increased observing to some degree, but only in high observers. Thus, in both cases, greater sensitivity to uncertainty seems to depend on individual tendencies. Greater reinforcement uncertainty did lead to more MBP, particularly on the active side. This demonstrates the sensitivity of human performance to reinforcement rates in keeping with previous human instrumental task performance (Reed, 2015; Shanks & Dickinson, 1991).

The introduction of punishment yielded a robust increase in observing, regardless of individual differences. This is consistent with the notion that checking escalates rapidly to avoid perceived or real aversive consequences. Under threat uncertainty, participants avoided the inactive side that led to punishment. This group did not differ in overall responding or reward rates, indicating the punishment did not influence overall motivation or task engagement as long as observing was available. Preliminary evidence from rodents indicates that punishment on the inactive side (foot shocks) also results in greater observing rates (Atherton et al., 2014). Upon the subsequent removal of the observing cue (see supplemental materials), participants under punishment now responded less and earned fewer rewards. This reduction was not noted in the other groups suggesting that when uncertainty is coupled with unavoidable aversive events, participants adopt an overall avoidant strategy.

The follow-up session enabled inspection for possible persistence in observing. Observing proved highly stable within individuals regardless of changes in uncertainty. Participants generally, continued to respond as before, observing style by session interaction: *F*(1, 71) = 1.77, *p* = .19. Moreover, those previously under punishment persisted with high observing levels and avoiding the inactive side, despite being informed that there was no longer any punishment and despite observing costs. These individuals still reported a drop in anxiety over the session. To investigate further, observing in this second session was binned into 2-min intervals. Observing for the formerly punished remained higher than the formerly baseline for four bins (*p*s < .05), dropping to baseline levels only in the final bin. Thus, participants demonstrated persistent observing and avoidance of the no-longer punished inactive side despite being informed that they would no longer be punished.

Performance of the previously punished group is reminiscent of the persistence reported in OCD patients when cues predicting shocks are devalued (Gillan et al., 2015; Gillan et al., 2014). Though a different task, participants are similarly informed that aversive consequences would no longer occur. Nevertheless, OCD patients continued to respond to avoid aversive shocks. Such behaviour has been interpreted as reflecting habitual responding rendering behaviour less sensitive to current goals. Present performance in healthy participants may be indicative of how easily inflexible responding can develop, being insensitive to changes in environmental contingencies. Avoidance behaviours can be particularly prone to habitual and rigid control (Gillan, Urcelay, & Robbins, 2016). Present results also dovetail with the finding that repeated checking specifically leads to automatisation of checking behaviour not only in patients but also in healthy individuals (Dek, van den Hout, Engelhard, Giele, & Cath, 2015). Thus, some excessive checking may be triggered from a once-appropriate high level of checking that does not subside due to habit or automaticity, even when the need for greater checking diminishes.

## Experiment 2

Given the robust effect of punishment, we sought to further investigate which aspects of threat uncertainty may influence observing. The outcome contingencies in Experiment 1 meant that though observing was functional and was associated with more rewards and fewer punishments, any translation into greater earnings was counteracted by the cost, albeit small, of the observing response. Here, we assessed whether a greater cost to reducing task uncertainty would influence observing. Furthermore, the outcome of an observing response had been fully predictable, always yielding the informative cue. One potential factor that may contribute to escalation of checking is whether the information gained does indeed reduce uncertainty. Checking may not suffice to alleviate uncertainty due to internal factors, such as doubt, and/or external factors, such as unreliable information. Therefore, we manipulated the reliability of the observing cue. Namely, participants had to press several times to attain the cue.

Compulsive checking is characterised not only by excessive checking but also by its non-functional nature. A key observation in the rodent ORT was the presence of “extra-observing button presses” (EOBP) whereby rats continued to observe despite the cue already being available. This rodent procedure involved an observing cue of 30 s and the administration of quinpirole, neither being feasible in the human procedure. Thus, EOBP proved rare in Experiment 1. We speculated that not only would making the cue more unreliable increase observing overall, but also that it might serve to increase non-functional observing.

### Method

#### Participants

Sixty participants (*M* = 23.87 years, *SD* = 6.01) were compensated at a rate of £8 per hour. All had normal or corrected-to normal vision and hearing, no psychiatric conditions and were not taking any psychotropic medication. The three groups did not differ in age, *F*(2, 57) = 1.49, *p* = .23, and were matched for gender (11 females per group).

#### Procedure and design

The procedure was based on the punishment condition in Experiment 1, with three between-subject conditions. FR1 (Fixed Ratio of 1, where every observing response yielded the cue) replicated the previous punishment condition, with observing costing 5 p. In increased cost, the observing schedule was FR1 but the cost for each observing response was threefold: 15 p. In VR3, the cost was 5 p, but the observing cue appeared every 3 responses on average (range 2-4). Additionally, no follow-up session occurred and questionnaires were completed at the session onset.

### Results

#### Observing stage

##### Observing

**Figure 3.**
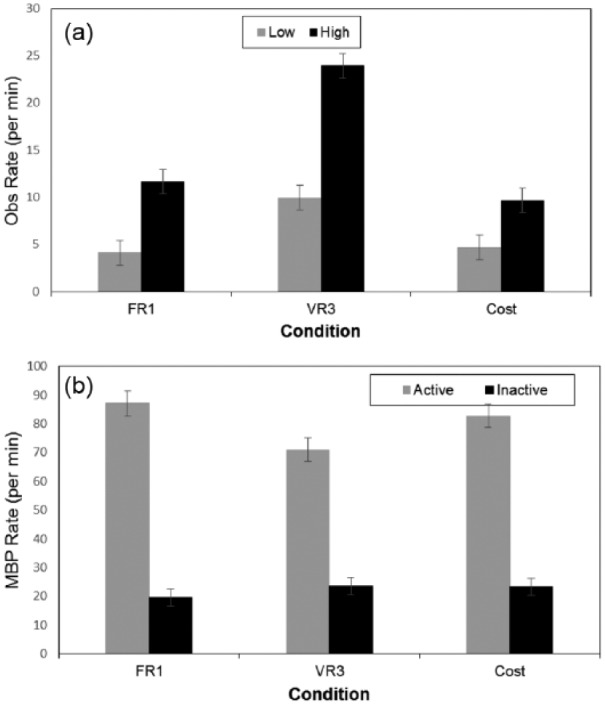
(a)Mean observing rate for high and low observers. (b) Mean button press for active and inactive sides in Experiment 2. OBS: observing; MBP: mean button press. Error bars depict the standard error of the mean.

An 3 x 2 ANOVA with condition and observing style revealed effects for condition, *F*(2, 54) = 35.39, *p* < .0001, η_p_^2^ = 0.57; observing style, *F*(1, 54) = 69.98, *p* < .001, η_p_^2^ = 0.56; and the interaction, *F*(2, 54) = 6.42, *p* = .003, η_p_^2^ = 0.19 (Figure 3). Observing style was determined by a median split within each condition (8.15, 16.93, and 8.00 for FR1, VR3, and increased cost, respectively). FR1 and increased cost did not differ, but in VR3 both low, *F*(1, 54) = 10.15, *p* = .002, and high observers, *F*(1, 54) = 44.91, *p* < .001, had greater observing rates compared to baseline low and high observers, respectively.

In contrast to FR1, not all observing responses under the VR3 condition yielded a cue. An analysis of successful observing responses comparing VR3 and FR1, revealed a difference, *F*(1, 36) = 9.28, *p* = .0043, η_p_^2^ = 0.21, whereby VR3 had reduced rates of successful observing compared to FR1. The interaction between condition and observing style was not significant, *F*(1, 36) = 2.97, *p* = .093.

We noted an increased frequency of extra-observing button presses (EOBP) in VR3. Only 15% of FR1, and 25% of increased cost, in contrast to 65% of the VR3 group had any EOBP, χ^2^(2) = 12.31, *p* = .002. Nevertheless, EOBP rates were still low.

##### MBP

A 3 x 2 x 2 ANOVA included condition, observing style and active versus inactive side as independent variables. Side was significant, *F*(1, 54) = 312.79, *p* < .001, η_p_^2^ = 0.85. The interaction between condition and side, *F*(2, 54) = 3.17, *p* = .0497, η_p_^2^ = 0.11, was due to lower responding in VR3 versus FR1 on the active side, *F*(1, 54) = 4.93, *p* = .031. There was an interaction between style and side, *F*(1, 54) = 22.14, *p* < .001, η_p_^2^ = 0.29, with a greater difference between active and inactive MBP for high versus low observers (for all other effects *p*s > .22).

##### Reward and punishment rates and earnings

A one-way ANOVA on rewards did not detect significant effects (*p*s > .12), though the planned comparison between FR1 and VR3 indicated reduced reward rates for the latter, *F*(1, 54) = 4.10, *p* = .048; *M* = 4.83, *SD* = 1.07 versus *M* = 3.86, *SD* = 1.53, respectively. An ANOVA on punishments indicated style was significant, *F*(1, 54) = 49.42, *p* < .001, η_p_^2^ = 48, with increased punishment in low, *M* = 1.48, *SD* = 0.88, compared with observers, *M* = 0.33, *SD* = 0.22. Remaining effects were non-significant (*p*s > .17). The ANOVA on earnings showed a condition effect, *F*(2, 54) = 10.36, *p* < .001, η_p_^2^ = 0.28. FR1 earned more, (*M* = £ 16.24, *SD* = 6.96) than VR3 (*M* = £ 5.51, *SD* = 7.69) or increased cost (*M* = £8.10, *SD* = 8.73), while the latter two did not differ (*p* > .29). Remaining effects were not significant (*p*s > .23).

#### Self-reported anxiety

A mixed ANOVA included condition, observing style and experiment stage. Anxiety increased as the session progressed: from *M* = 7.60, through *M* = 9.42 to *M* = 11.67, *F*(2, 108) = 16.60, *p* < .001, η_p_^2^ = 0.239.

#### Questionnaires

Self-reported depression, IU, OC symptoms, and anxiety did not correlate with task measures. Age correlated negatively with MBP, *r* = –.30, *t*(58) = 2.44, *p* = .018, and with earnings, *r* = –0.42, *t*(58) = 3.51, *p* < .001. Mean VAS anxiety correlated positively with STAI state, *r* = 0.51, *t*(77) = 4.46, *p* < .001; trait, *r* = .47, *t*(77) = 4.10, *p* < .001; OCI-R, *r* = 0.28, *t*(77) = 2.22, *p* = .030; and IU, *r* = .44, *t*(58) = 3.71, *p* < .001. There were no group differences in questionnaire measure (*p*s > 0.17).

### Summary of results

Performance did not appear to be particularly sensitive to the cost of observing, with no significant differences between FR1 and cost groups despite a threefold difference in observing expense. VR3 led to greater observing rates, but closer to a two-fold rather than a three-fold increase, leading to overall fewer observing cues. This meant less reduction of uncertainty and more punishments, which coupled with the presence of unsuccessful observing meant less earning. Nevertheless, MBP remained stable as increased punishment did not lead to greater avoidance. VR3 was also associated with more EOBP, although these remained infrequent overall. Given that observing ceased when the cue was extinguished, participants largely observed in a goal-directed manner.

### Discussion

The results indicated that a threefold increase in the cost of observing, be it in effort or in earnings, did not deter participants from checking. Individuals in the increased cost condition behaved similarly to FR1 and seemed insensitive to the observing cost, resulting in reduced earnings. This reinforces the notion that observing levels appear relatively stable, with cost, at least with present parameters, playing a minor role. This is reinforced by the similar performance in FR1 in this and the previous experiment. At the same time, individuals in increased cost did perform better compared with the FR1 group when the observing cue was removed (see supplemental materials). Possibly, these individuals learned more from the punishment because the loss of points in the observing stage was more salient. Future studies should examine how the value matrix and its change over time may influence performance.

A threefold increase in effort to observe yielded greater observing regardless of observing style. However, this was not a threefold increase, and this group did not achieve the same level of successful observing. The resulting greater uncertainty was associated with less efficient allocation of resources with reduced active side responding so these participants earned fewer rewards. This may be due to ceiling effects and points again to the relative consistency of observing rates in the current procedure. Thus, at least in healthy volunteers, although observing increases with external unpredictability, there is a limit on how much it will escalate. This is consistent with the infrequent dysfunctional checking and the refrain from observing responses when they no longer served to reduce uncertainty.

## Experiment 3

The previous experiments point to the persistence and relative individual stability of checking levels. Here, we asked whether individuals escalate their already established observing responding when the consequences of uncertainty change. Checking and reassurance seeking presumably escalate with increased threat uncertainty and the desire to avoid perceived aversive consequences (Grupe & Nitschke, 2013). Specifically, we assessed whether individuals adjust their observing with the introduction of punishment. This was particularly of interest in low observers as this might provide insight into protective attitudes and behaviours in the face of uncertainty. Previous experiments indicated that low observers persist in this pattern of responding, enduring greater uncertainty and more punishments. A recurring theme emerged during debriefing, of low observers not minding the uncertainty and expressing a desire to not rely on external cues available. Thus, here we examined whether punishment, introduced after a baseline period, would lead to the escalation of observing and avoidance of the inactive side for all individuals.

### Method

#### Participants

Twenty participants (17 female) (*M* = 24.14 years, *SD* = 2.90) took part and compensated at a rate of £8 per hour. All had normal of corrected-to normal vision and hearing, no psychiatric conditions and were not taking any psychotropic medication.

#### Procedure and design

All participants performed the baseline condition followed by punishment (FR1). All other aspects were as in Experiment 1 with questionnaires administered at the onset. Questionnaire data are reported in Experiment 4 to increase power for those analyses. A manipulation check verified that all participants perceived the punishment as more aversive.

### Results

#### Observing stage

##### Observing

**Figure 4.**
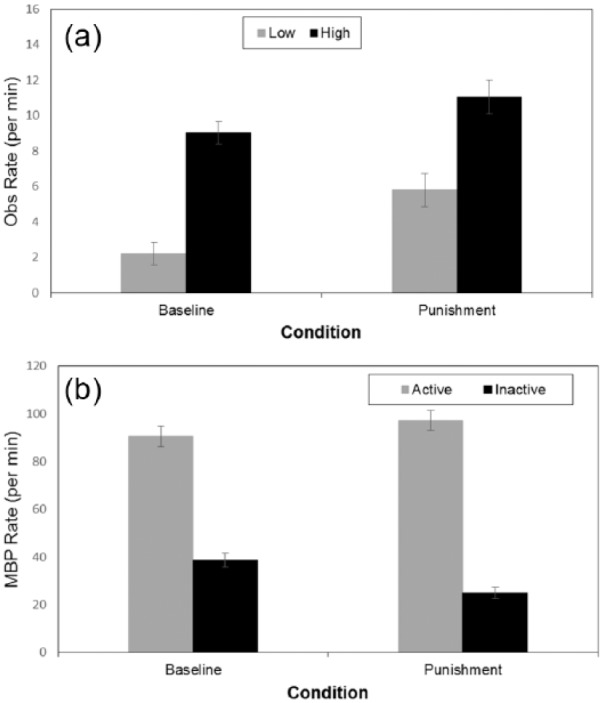
(a) Mean observing rate for high and low observers. (b) Mean button press for active and inactive sides in Experiment 3. OBS: observing; MBP: mean button press. Error bars depict the standard error of the mean.

A 2 x 2 two-way ANOVA was conducted with observing style and condition (baseline/punishment), with the latter a within-subjects variable (Figure 4). Median split at baseline determined observing style (Md = 5.73), with greater levels for high observers, *F*(1, 18) = 54.42, *p* < .001, η_p_^2^ = 0.75. There was an effect for condition, *F*(1, 18) = 13.39, *p* = .002, η_p_^2^ = 0.43, with greater observing in punishment compared with baseline. The interaction was not significant, suggesting observing increased regardless of style (*p* = .30).

##### MBP

A 2 x 2 x 2 ANOVA included observing style as a between-subjects variable and condition and side (active versus inactive button) as within-subject variables. MBP were higher on the active versus inactive side, *F*(1, 18) = 201.76, *p* < .001, η_p_^2^ = 0.92. There was an interaction between condition and side, *F*(1, 18) = 13.34, *p* = .0018, η_p_^2^ = 0.43, with the difference between the active and inactive becoming larger under punishment. There was both increased active MBP, *F*(1, 18) = 5.73, *p* = .028, and decreased inactive MBP, *F*(1, 18) = 12.46, *p* = .002. There was also a side by observing style interaction, *F*(1, 18) = 25.98, *p* < .0001, η_p_^2^ = 0.59, with a greater difference for side in high versus low observers. This was due to both greater active MBP, *F*(1, 18) = 9.79, *p* = .006, and reduced inactive MBP in high observers, *F*(1, 18) = 26.37, *p* < .001. Finally, an interaction between condition and style, *F*(1, 18) = 7.75, *p* = .012, η_p_^2^ = .30, showed that low observers responded less overall in punishment compared with baseline, *F*(1, 18) = 10.75, *p* = .004, but high observers did not, *F*(1, 18) = 0.43, *p* = .518. The three way interaction was not significant (*p* = 0.183).

#### Reward, punishment and earnings

There was an effect of style on rewards, *F*(1, 18) = 9.44, *p* = .007, η_p_^2^ = 0.34, with greater rewards in high (*M* = 5.91, *SD* = 0.77) versus low observers (*M* = 4.39, *SD* = 1.36). Condition was not significant, *p* = .093, nor was the interaction, *p* = 0.61. During punishment, high observers experienced fewer punishments (*M* = 0.44, *SD* = 0.45) compared with low observers (*M* = 1.23, *SD* = 0.67), *F*(1, 18) = 9.55, *p* = .006, η_p_^2^ = 0.34.

As expected from these results, high observers earned more (*M* = £23.44, *SD* = 4.64) than low observers (*M* = £17.00, *SD* = 6.23), *F*(1, 18) = 6.89, *p* = .017, η_p_^2^ = 0.27. The ANOVA on earnings also indicated condition was significant, *F*(1, 18) = 75.05, *p* < .001, η_p_^2^ = 0.81, with greater baseline earnings (*M* = £24.03, *SD* = 5.98) compared with punishment (*M* = £16.41, *SD* = 7.41). The interaction was significant, *F*(1, 18) = 10.50, *p* = .005, η_p_^2^ = 0.37, with an effect for observing style with punishment, *F*(1, 18) = 12.74, *p* = .002, but not baseline, *F*(1, 18) = 1.89, *p* = .186.

##### Self-reported anxiety

An ANOVA with stage, condition and observing style revealed an effect for condition, *F*(1, 18) = 51.31, *p* < .001, η_p_^2^ = 0.74, with greater anxiety in punishment (*M* = 13.52, *SD* = 7.20) compared with baseline (*M* = 8.65, *SD* = 5.89). Anxiety also increased across stages within each condition, *F*(2, 36) = 17.33, *p* < .001, η_p_^2^=0.49. There was also an interaction, *F*(2, 36) = 5.52, *p* = .008, η_p_^2^ = 0.23, such that anxiety rose more within punishment compared with baseline.

### Summary of results

Introduction of punishment led to a robust rise in observing in participants regardless of observing style. Furthermore, participants used the cue and avoided the inactive side, focusing their efforts on the side yielding rewards. Unavoidable punishment in the post observing stage conveyed information allowing participants to favour the active side but also led to less responding overall (see supplemental materials). Though observing style was a secondary factor, we noted that low observers made fewer MBP and did not seek as much information to reduce the uncertainty and so received fewer rewards and more punishments compared with high observers. All this served to make them earn less overall in punishment. Anxiety levels increased as the task progressed, with the introduction of punishment and then with the removal of the observing cue and resulting greater punishment rates.

### Discussion

The main findings indicate that the introduction of threat uncertainty, as implemented by punishment, resulted in greater observing not only across individuals (Experiment 1) but also within individuals. Hence, individuals are sensitive to changes in the consequences of uncertainty with checking behaviours escalating to avoid anticipated threat. Regardless of initial observing levels, punishment had a robust effect, with participants avoiding the inactive side. This is even more striking given that this approach was intuitively adopted by participants without any instructions. While low observers checked more under punishment, they consistently observed less than the high observers. Consequently, they were less effective in concentrating their efforts on the active side and avoiding the punished side, receiving more punishments and earning less overall. Thus, those who engaged in greater observing initially, benefited from this strategy under punishment, demonstrating the utility of observing within current parameters.

## Experiment 4

The first three experiments served to validate the ORT and provide information about the role of anticipated threat in the escalation and maintenance of checking behaviours. A key prediction emerging from the evidence thus far, would be that individuals with OCD, who are characterised by excessive doubt and IU (Holaway et al., 2006; Tolin et al., 2003), would adopt a strategy similar to that of high observers. Given that OCD patients exhibit extensive avoidant behaviours (Ettelt et al., 2008) and are presumably hyper-sensitive to threat uncertainty (Grupe & Nitschke, 2013), the introduction of punishment could possibly serve to escalate observing excessively in this group. To assess these predictions, we tested OCD patients and a control group using the same procedure as in Experiment 3.

### Method

#### Participants

Twenty-one OCD patients and 21 healthy controls participated (see Table 1). Patients met *Diagnostic and Statistical Manual of Mental Disorders* (DSM-IV) criteria for OCD but not other axis-I disorders as determined by a detailed structured clinical interview with a psychiatrist. All but one patient were prescribed serotonin reuptake inhibitors with four receiving an adjunct antipsychotic, one an adjunct antidepressant and one receiving both, and another prescribed pregabalin. One patient was also prescribed lithium carbonate. Controls had no current or past psychiatric disorders as determined by a screening interview including the Mini International Neuropsychiatric Interview (MINI; Sheehan et al., 1998), and were not taking any psychoactive medications. For all participants, exclusion criteria included current or past neurological disorders or brain damage.

**Table 1.** Means and standard deviations of control and OCD patient group characteristics.

Characteristic	Measure	Controls (*n* = 21)	OCD (*n* = 21)	*t*	*p*
		*M* (*SD*)	*M* (*SD*)		
Age	Years	39.1 (11.9)	45.2 (12.8)	1.5	.11
Gender	M:F	10:11	10:11		
Verbal IQ	NART	112.2 (7.0)	115.4 (5.6)	1.4	.15
Years	Education	13.8 (1.8)	13.9 (1.9)	0.3	.81
Obsessions and Compulsion	YBOCS	0.0 (0.2)	22.43 (5.7)	18.0	<.001
Depression	MADRS	2.8 (3.1)	8.5 (4.7)	4.7	<.001
Depression	BDI				
State Anxiety	STAI-S	33.2 (10.2)	49.6 (11.3)	4.9	<.001
Trait Anxiety	STAI-T	38.0 (10.5)	63.1 (8.9)	8.3	<.001
IU	IU				
Obsessions and Compulsion	OCI-R	10.8 (9.6)	34.8 (9.4)	8.2	<.001

NART: National Adult Reading Test; YBOCS: Yale-Brown Obsessive Compulsive Scale; BDI: Beck Depression Inventory; STAI-S: State/Trait Anxiety Inventory-State; STAI-T: State/Trait Anxiety Inventory-Trait; IU: Intolerance of Uncertainty; OCI-R: Obsessive Compulsive Inventory-Revised.

#### Design, procedure and apparatus

The experiment was identical to Experiment 3 with the following exceptions. The design included group (OCD, controls) and observing style (low, high observing) as between-group variables and condition (baseline, punishment) as a within-subject variable. Prior to testing, symptom severity was assessed with the Yale-Brown Obsessive-Compulsive Scale (YBOCS; Goodman et al., 1989). Verbal IQ was assessed with the National Adult Reading Test (NART; Nelson, 1982) and depression symptom severity was assessed with the Montgomery-Asberg Depression Rating Scale (MADRS; Montgomery & Asberg, 1979). At the end, all participants reported perceiving the punishment as aversive. Participants performed additional tasks not reported here. The experiment was run on a 17.3″ laptop with connected mouse, running Windows 7. The Cambridge Local Research Ethics Committee (08/H0308/65) approved the study.

### Results

#### Observing stage

##### Observing

**Figure 5.**
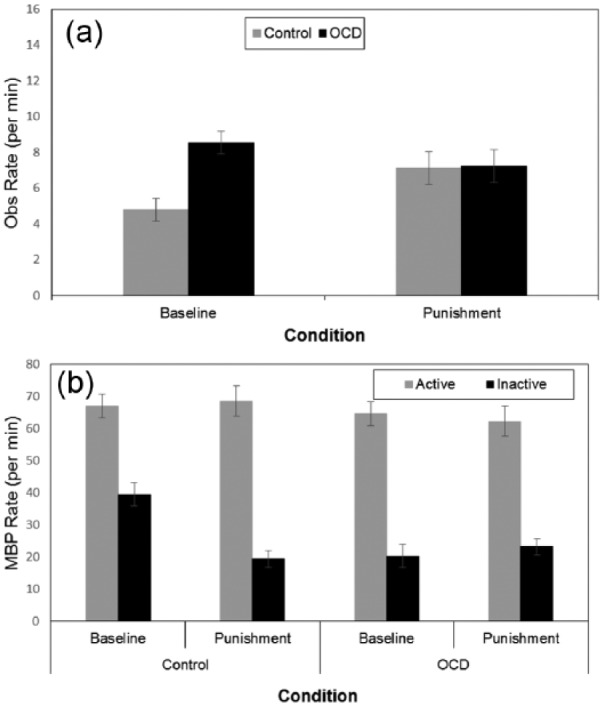
(a) Mean observing rate for the control and OCD group. (b) Mean button press for active and inactive sides for the two groups in Experiment 4. OBS: observing; MBP: mean button press; OCD: obsessive compulsive disorder. Error bars depict the standard error of the mean.

A 2 x 2 x 2 three-way mixed ANOVA revealed effects for group, *F*(1, 38) = 5.19, *p* = .028, η_p_^2^ = 0.12, and observing style, *F*(1, 38) = 39.39, *p* < .001, η_p_^2^ = 0.51, but not condition, *F*(1, 38) < 1, *p* = .47. Observing median split indicated medians of 4.15 and 7.7 for controls and patients, respectively. All two-way interactions were significant though the three-way interaction was not, *F*(1, 38) < 1, *p* = .99. The group by condition, *F*(1, 38) = 6.35, *p* = .016, η_p_^2^ = 0.14, indicated greater observing rates in patients compared with controls in baseline, *F*(1, 38) = 17.44, *p* < .001, but not punishment, *F*(1, 38) < 1, *p* = .92, (Figure 5). Secondary comparisons assessed the finding of increased observing with punishment was replicated in each group. This was found in controls, *F*(1, 38) = 5.25, *p* = .027, η_p_^2^ = 0.20, but not patients who showed a non-significant decrease, *F*(1, 38) = 1.62, *p* = .21, η_p_^2^ = 0.06. The group by observing style, *F*(1, 38) = 5.13, *p* = .029, η_p_^2^ = 0.12, indicated no difference in low observers, *F*(1, 38) < 1, *p* = .99, but increased rates in patients compared with controls in high observers, *F*(1, 38) = 4.44, *p* = .042. The observing style by condition, *F*(1, 38) = 8.90, *p* = .005, η_p_^2^ = 0.19, stemmed from greater observing in punishment only in low observers.

##### MBP

A four-way 2 x 2 x 2 x 2 mixed ANOVA revealed effects for side, *F*(1, 38) = 188.31, *p* < .001, η_p_^2^ = 0.83, and condition, *F*(1, 38) = 17.42, *p* < .001, η_p_^2^ = 0.31. There were two-way interactions between condition and group, *F*(1, 38) = 19.60, *p* < .001, η_p_^2^ = 0.34, condition and observing style, *F*(1, 38) = 19.20, *p* < .001, η_p_^2^ = 0.33, and observing style and side, *F*(1, 38) = 20.24, *p* < .001, η_p_^2^ = 0.34. Finally, there was a three way interaction between condition, group and side, *F*(1, 38) = 9.09, *p* = .005, η_p_^2^ = 0.19. This stemmed from the controls responding more on the inactive side under baseline. With the introduction of punishment, controls avoided the now punished side responding similarly to patients.

##### Reward, punishment and earnings

The three-way mixed ANOVA on reward rates revealed no significant effects (*p*s > .22) with mean reward rates being 3.64 (*SD* = 1.39) and 3.23 (*SD* = 1.39) for controls and OCD patients, respectively. An ANOVA on punishment rates revealed only an effect for observing style, *F*(1, 38) = 7.12, *p* = .011, η_p_^2^ = 0.16, with greater punishment for low, (*M* = 1.31, *SD* = 0.86) compared with high observers (*M* = 0.62, *SD* = 0.82). The ANOVA on earnings showed an effect of condition, *F*(1, 38) = 45.35, *p* < .001, η_p_^2^ = 0.54, with reduced earnings in punishment (*M* = £8.64, *SD* = 8.40) compared with baseline (*M* = £14.30, *SD* = 7.53). There was also an interaction between condition and observing style, *F*(1, 38) = 16.83, *p* < .001, η_p_^2^ = 0.31, such that high- earned more than low observers during baseline but not punishment, presumably due to increased observing in the latter group.

#### Self-reported anxiety

An ANOVA with group, observing style, condition, and stage revealed effects for group, *F*(1, 38) = 13.83, *p* < .001, η_p_^2^ = 0.27, with greater anxiety in patients (*M* = 12.99, *SD* = 7.00) compared with controls (*M* = 5.79, *SD* = 5.50). Additionally, there was an effect for stage, *F*(2, 76) = 15.18, *p* < .001, η_p_^2^ = 0.28, with anxiety levels being 8.28, 9.23, and 10.66 for measurements 1 through 3, respectively (see Figure 1f). Condition was also significant, *F*(1, 38) = 4.25, *p* = .046, η_p_^2^ = 0.10, with levels higher in punishment (*M* = 9.83, *SD* = 7.18) compared with baseline (*M* = 8.95, *SD* = 7.22). Finally, there was an interaction between group, observing style and stage, *F*(2, 76) = 6.26, *p* = .003, η_p_^2^ = 0.14, resulting from increased anxiety in high observing patients following removal of the observing cue, *F*(1, 38) = 17.15, *p* < .001.

#### Questionnaires

Data regarding associations between task performance and questionnaires were combined for Experiments 3 and 4 to increase power, as they employed the same procedure (see Table 2). Baseline observing correlated positively with trait anxiety. Baseline MBP correlated negatively with all questionnaires. Similarly, punishment MBP correlated negatively with OC symptoms, depression, and anxiety, as did earnings. As in Experiment 2, age was negatively associated with MBP and with earnings. VAS anxiety correlated positively with all questionnaire self-reported measures (see Table 2). We further tested whether OCI checking would correlate with baseline observing. There was a significant, albeit modest, correlation, *r* = 0.30, *t*(60) = 2.41, *p* = .019. Given the importance of IU, we assessed the two subscales and noted a significant correlation between baseline observing and factor 2: “uncertainty is unfair and spoils everything” (Sexton & Dugas, 2009), *r* = .32, *t*(60) = 2.62, *p* = .011. However, any interpretation must consider the exploratory nature of this analysis.

**Table 2.** Correlations between task performance and individual characteristics for all participants in Experiments 3 and 4.

	Age	Depression	Intolerance of Uncertainty	OC symptoms	State anxiety	Trait anxiety
Observing-baseline	.17	.20	.23	.23	.20	.27*
Observing-punishment	–.11	.07	–.00	–.11	.04	.00
MBP-baseline	–.41**	–.30*	–.31*	–.43***	–.33**	–.39**
MBP-punishment	–.42**	–.24	–.20	–.27*	–.26*	–.25
Earnings	–.41**	–.26*	–.21	–.36**	–.33*	–.31*
VAS	.03	.50***	.44**	.44***	.59***	.49***

MBP: main button press; VAS: mean visual analogue scale.

* *p* < .05, **p < .01, ***p < .001.

#### Summary of results

OCD patients exhibited increased baseline observing and, unlike controls, did not adjust this with the introduction of punishment. With higher observing during baseline, the patients already avoided the inactive side despite no aversive consequences. When punishment was introduced controls now observed like patients, while patients persisted in the same pattern. Overall, OCD patients did not obtain more rewards nor did they earn more than controls and their greater baseline observing did not translate into a more effective response strategy. When punishment was introduced controls avoided it to the same extent. Thus, excessive avoidance of the inactive side in patients did not entail increased active side responding. For all participants, anxiety rose following the observing stage, increasing further with the removal of the observing cue. OCD patients reported feeling more anxious throughout compared with controls, with removal of the cue resulting in even greater anxiety.

Observing levels were somewhat preserved across the task, with a moderate association between baseline and punishment, *r* = 0.44, *t*(61) = 3.87, *p* < .001. Questionnaire data yielded several observations: (a) older participants pressed at slower rates and earned less; (b) increased trait anxiety was associated with greater baseline observing, with OC symptoms, IU and negative mood not reaching significance although being positively correlated; and (c) higher levels of OC symptoms, IU, and negative mood were all associated with reduced MBP responding both during baseline and punishment, thereby being associated with poorer earnings.

### Discussion

This experiment showed that OCD patients indeed demonstrate elevated levels of certainty seeking in the ORT. Contrary to the prediction, punishment did not lead them to seek even greater certainty. While controls increased observing to avoid the inactive and now punished side, patients appeared insensitive to the introduction of threat uncertainty. Possibly, patients were at ceiling. However, even higher observing levels were noted in VR3 in Experiment 2, precluding this notion. It is more likely that once patients adopted a particular strategy, this was not easily adapted to a changing environment, indicative of the general inflexibility often characterising OCD (Snyder et al., 2015). It is further possible that impaired response control under punishment, presently manifested in this rigid and abnormal strategy (Morein-Zamir et al., 2013). The results from the main button instrumental responding indicate that not only did patients observe more at baseline, but also that they used this information to avoid the inactive side despite limited negative consequences. Nevertheless, their increased observing did not translate into greater earnings.

Controls who observed more were shown here as in Experiment 3 to use this information to gain more rewards. High observing in patients, however, did not translate into more active side responding. This points to a potential dissociation between the tendency in patients to seek certainty and their ability to use this information effectively. Thus, although one could have anticipated an advantage to greater certainty seeking, at least in terms of rewards gained, this was not the case. This is reminiscent of previous findings where unlike controls, OCD patients failed to adjust their checking in response to external error signals (Rotge et al., 2015), further pointing to rigid, non-functional certainty seeking in OCD.

This study replicated the finding of greater observing with the introduction of threat uncertainty in an older community sample of healthy individuals, as was found between groups (Experiment 1) and within participants (Experiment 3). We noted an interaction between response style and condition, not evidenced in Experiment 3. However, high observers comprised both patients and controls and the three-way interaction was not significant suggesting caution in interpreting any role of response style. Consideration of response style does however point to individual differences in this construct even in the patients.

## General discussion

This study assessed checking behaviours and contributors to compulsive checking in humans, using a free operant ORT previously validated in rats (Eagle et al., 2014). Checking was operationalised as observing responses whereby individuals could reduce uncertainty, and gain information about the task. The results indicated that the ORT is translational, showing similar patterns of behaviour in rodents and humans. Experiment 1 showed that threat uncertainty leads to increased certainty seeking which persists even when the threat is removed. Experiment 2 revealed that with the outcome contingencies employed, participants appear insensitive to initial checking costs and similarly a threefold increase did not deter participants from observing, though to a lesser extent. Experiment 3 confirmed individuals would escalate their checking when threat uncertainty is introduced, while Experiment 4 revealed elevated observing levels in OCD patients, who were abnormally rigid when punishment was instated.

The ORT offers a unifying method for cultivating interactions between research approaches. Theoretical constructs from different levels of analyses can be integrated to better understand compulsive checking and certainty seeking. Psychological concepts believed to contribute to the development of compulsive checking, such as IU, may be coupled with cognitive constructs such as impaired response inhibition and excessive rigidity (Linkovski, Kalanthroff, Henik, & Anholt, 2015; Morein-Zamir, Fineberg, Robbins, & Sahakian, 2010). Compulsive certainty seeking may be further exacerbated and maintained by weakened or weakening goal-directed control in the face of perceived or anticipated threat uncertainty, possibly manifested by beliefs such as enhanced responsibility (Gillan et al., 2014; Grupe & Nitschke, 2013; Ladouceur, Rhéaume, & Aublet, 1997; Lind & Boschen, 2009). The ORT could promote formal assessment of psychological concepts in ways amendable to brain imaging (in humans), lesions (in rodents), and pharmacological manipulations (in both).

### Relevance for translational models of compulsive checking and learning theory

Present findings offer further validity to the quinpirole-induced OCD animal model, as developed in the ORT (Eagle et al., 2014). Specifically, purposeful use of the observing cue was found in both rodents and humans, with the task capturing individual variability in observing in all populations examined. Inflexible observing was evidenced in the quinpirole-treated rats which were unresponsive to increased variability of response requirements on the active lever. Though we did not perform this manipulation in patients, they exhibited inflexible responding when threat uncertainty was introduced. The behavioural rigidity may, as in rats, be associated with disruption of frontostrital dopamine (Floresco, 2013). An important difference was the prevalence of non-functional observing in the rats, which was rarely seen in humans. Notably, for the rats no cost was associated with observing, while here a small cost (10% of reward magnitude) was present and observing cue duration was considerably briefer (1.5 s versus 15 s). Nevertheless, some EOBP was noted (Experiment 3) suggesting such non-functional responding can be elicited in humans.

The present procedure employed graded yet brief training, with participants only told to earn as much as possible. The latter is line with the rodent ORT where animals are required to earn rewards. This differs considerably from human observing research which typically employs complex idiosyncratic scenarios (Case & Fantino, 1989). Minimal instruction may be particularly beneficial in tasks where simpler mechanisms found in rodents could account for behaviours previously explained by higher order constructs, such as confidence and meta-cognition (Kepecs & Mainen, 2012; Kepecs, Uchida, Zariwala, & Mainen, 2008). The present procedure can nevertheless, be adapted to incorporate OCD-related themes such as enhanced responsibility (Arntz, Voncken, & Goosen, 2007; Ladouceur et al., 1997).

The learning literature has pointed to multiple factors seemingly contributing to observing behaviour (Case & Fantino, 1989; Shahan & Cunningham, 2015). The conditioned-reinforcement account focuses on the cue having conditioned reinforcing properties via association with positive reinforcement (Fantino, 1998; Fantino & Silberberg, 2010). The uncertainty reduction hypothesis focuses on the information provided by the cue (Lieberman, Cathro, Nichol, & Watson, 1997). This has been complicated by the notion that response efficiency (or utility) and uncertainty reduction *per se* are also potentially reinforcing (Fantino & Silberberg, 2010; Lieberman et al., 1997). The two-lever paradigm employed here, which ensures that unlike in previous observing studies, participants are continuously on task, may further clarify contributors to observing. In present form observing (a) served to reduce uncertainty, (b) provided useful information, and (c) likely acquired secondary reinforcer status. Notably, the ORT has the advantage of employing these multiple contributors to checking as found in everyday life, thereby increasing its external validity. Investing distinct contributions of uncertainty reduction versus any reinforcing properties of checking would elucidate how maladaptive checking develops.

### Relevance for cognitive and neuropsycholoigcal models of compulsive checking

The ORT provides an alternative approach to current certainty seeking procedures, which typically involve deciding whether to go back and verify a previously seen stimulus or reporting some aspect following multiple checks (Harkin & Kessler, 2009; Kim et al., 2012; Rotge et al., 2008). Observing does not rely on explicit memory or confidence in one’s memory, is unlikely to involve visuospatial difficulties or be driven by stimuli idiosyncrasies. Aberrant checking as noted in OCD patients here may be independent of these processes, adding to the mixed support for their involvement in compulsive checking (Clair et al., 2013; Cuttler & Graf, 2009). Excessive doubt, reliably seen in memory (Muller & Roberts, 2005), likely extends to other cognitive domains, and may have contributed to present OCD performance.

Self-reported anxiety levels tended to rise with task progression, particularly when participants experienced punishment but also during baseline. The latter effect could be attributed to a persistent state of uncertainty (De Putter, Van Yper, & Koster, 2017). Observing levels were moderately associated with anxiety in Experiments 3-4 where anxiety was not range restricted (see also supplemental materials). With the removal of the observing cue, anxiety increased in patients but not controls. This was noted even without the threat of punishment, further reinforcing the link between uncertainty and anxiety. There was also an association of observing with self-reported checking and aspects of IU, though an independent replication of this is warranted. The association between anxiety and observing emphasises the trans-diagnostic nature of certainty seeking (Boelen & Carleton, 2012). Furthermore, elevated observing was noted in the OCD patients, although they were not selected for checking compulsions. Instances of checking, such as reassurance seeking may also manifest in increased observing, given that observing can be a coping strategy fostering a sense of control (Coleman et al., 2011; Parrish & Radomsky, 2010; Starcevic et al., 2012). In rodents, there was no clear relationship between anxiety, such as elevated plus maze performance, and ORT measures (Eagle et al., 2014). However, the quinpirole model of OCD, manipulating striatal dopamine, does not appear to be related to anxiety (Vorhees, Johnson, Burns, & Williams, 2009). Serotonergic manipulations in the ORT may uncover such relationships given the efficacy of SSRIs in both OCD and anxiety disorders.

Reduced instrumental responding (MBP) was associated with increased self-reported OC symptoms, depression and anxiety, particularly prior to the introduction of punishment. This suggests a general avoidant strategy, though one that did not extend to observing, possibly due to its reinforcing properties. The revised reinforcement sensitivity theory (Gray & McNaughton, 2000) can accommodate the seemingly opposing pattern of associations. In this framework OCD and anxiety symptoms follow from overactivity of the checking mode in the behavioural inhibition system (BIS), which together with the Fight-Flight-Freeze System (FFFS) can lead to avoidance behaviours, such as suppression of the prepotent instrumental responses. Increased sensitivity to ambiguous cues, involving overactive FFFS and BIS (Heym, Kantini, Checkley, & Cassaday, 2015), would also underlie IU with the BIS eliciting checking as a defensive approach.

In any case, caution should be used when relating behavioural indices with retrospective self-report measures, in healthy volunteers but particularly in patients where insight may be compromised. Furthermore, as demonstrated in Experiment 1, self-report of traits and of long-term behaviours can be biased by situational variables such as recent experience of punishment.

### Strengths, limitations and future directions

This study presents a novel approach to certainty seeking, investigating behavioural indices and self-report measures in healthy volunteers and OCD patients with no comorbidities. The patients were chronically medicated and the role of medication, particularly SSRIs, should be explored. Additionally, the patients were not selected for checking compulsions and any overlap between everyday compulsive checking and observing remains to be established. Nevertheless, as proposed above, observing may capture a broader range of certainty seeking behaviours such as compulsive washing and reassurance seeking. Experiment 4 did not include a clinical control group, which would clarify whether the increased yet rigid observing is specific to OCD, although given the transdiagnostic nature of certainty seeking, this may not be the case. Future research should assess observing in relation to specific compulsions and to anxiety disorders.

Examination of healthy individuals can help elucidate the conditions under which certainty seeking increases, and how compulsive aspects may develop or be maintained. Thus, for example, it appears observing escalates easily, as evidenced with the introduction of punishment (Experiments 3 and 4). But once established, observing is difficult to reduce, even if the environment has changed and it no longer serves the same functional role (Experiment 1). Only a weak association between observing and self-reported measures was noted (see also Harkin & Kessler, 2009), possibly due to range restriction as participants were screened, and/or from non-clinical checking involving different causes and serving a function role, such as to counteract impaired working memory. Care must be taken when generalising between non-clinical and clinical samples as seemingly excessive or abnormal behaviour in non-clinical samples may arise through different mechanisms altogether (Cuttler & Graf, 2009). Even in clinical samples, compulsive checking is promoted by multiple psychological, cognitive, and situational variables.

The complex behaviour of free-operant responding may also avail itself to sophisticated analyses and hypothesis-driven computational modelling, already advancing the understanding of decision making in uncertain and ambiguous situations (Kepecs & Mainen, 2012; Kiani, Corthell, & Shadlen, 2014). Observing levels appeared stable within individuals. It remains to be determined whether this extends over longer durations and whether it is associated with everyday certainty seeking behaviours rather than retrospective self-report.

To conclude, this study supports the translational value of the ORT, which appears promising for the investigation of the neuropharmacological and neural basis of compulsive checking.

## Supplementary Material

Supplementary material
