# Supplementary material for: Free operant observing in humans: a translational approach to compulsive certainty seeking
Source: Q J Exp Psychol (Hove). 2018 Jan 1;71(10):2052–69. doi: 10.1177/1747021817737727 (PMC6159779; doi:10.1177/1747021817737727)
Supplement: Supplementary material [file QJE-STD_17-067.R2-Supplemental_Material.docx]

**Supplemental Material for**

**Free operant observing in humans: a translational approach to compulsive certainty seeking**

Sharon Morein-Zamir, Sonia Shahper, Naomi A Fineberg, Verena Eisele, Dawn M Eagle, Gonzalo Urcelay, Trevor W Robbins

Additional Analyses

Below are analyses of performance during the post observing stage where, though observing responses were monitored, they did not lead to the cue. To summarize, in all experiments participants acted in a goal-directed manner in accordance with instructions, and ceased observing when the cue was removed.

Associations are also presented between self-report measures and baseline MPB and observing rates, in low and in high observing individuals separately (we thanks an anonymous reviewer for suggesting these analyses). To summarize, the pattern of associations reported in the main text for Experiments 3 and 4 was found within each sub-group. A similar pattern of associations was found for low observers in Experiment 1. The presence of punishment in Experiment 2, which led to greater observing levels overall, likely precluded these associations from emerging. Future studies may investigate the robustness of the associations at baseline given the moderate effect size and that high observers in Experiment 1 do not show similar results.

**Experiment 1**

***Observing.*** Over 92% of participants made no observing responses, with the remaining pressing rarely and likely inadvertently.

***MBP.*** An ANOVA with condition, side and observing style indicated an effect for condition (*F*(3, 71)=5.12,*p*=.003,η_p_^2^=0.18). *Post-hoc* analyses revealed reduced responses under punishment (M=59.12,SD=11.38) compared to baseline (M=61.62,SD=14.06), with no difference between baseline, increased effort (M=71.64,SD=11.21) and increased unpredictability (M=70.54,SD=12.21). Side was not significant (*F*(1, 71)=.01,*p*=.923) indicating that without the observing cue, participants were unable to determine reliably the active side. MBP rate was 65.86 (SD=18.81) and 65.60 (SD=17.41) for active and inactive sides, respectively.

***Reward, punishment and earnings.*** An ANOVA with condition and observing style on reward rate indicated condition was significant (*F*(3, 71)=12.64, *p<*.001, η_p_^2^=0.35). *Post-hoc* analyses revealed reduced rewards for increased effort (M=1.42, SD=0.73) and for punishment (M=2.35,SD=1.21) compared to baseline (M=3.51,SD=1.64), but no difference between the latter and increased unpredictability (M=3.41,SD=1.10). No other effects reached significance (p>.5 for all). Similarly, the ANOVA on earnings showed only a condition effect (*F*(3, 71)=28.35, *p*<.001, η_p_^2^=0.54). *Post-hoc* analyses indicated reduced earnings for increased effort (M=2.82,SD=1.45) (M=6.99,SD=3.28) and punishment (M=1.12,SD=2.33) compared to baseline, but no difference between the latter and increased unpredictability (M=6.80,SD=2.18). Under punishment, there was no difference for punishment between low (M=1.66, SD=1.15) and high (M=1.90,SD=0.27) observers (*F*(1, 18)=.45,*p*=.509).

***Questionnaires.*** The results are shown in Table S1.

**Experiment 2**

***Observing.*** No participants observed in FR1 and only 4 in VR3 and in increased cost, though rarely.

***MBP.*** There were no effects for condition or observing style, but responding was greater for active (M=71.12,SD=23.60) versus inactive side (M=51.75,SD=13.44; *F*(1, 54)=31.71, p<.001, η_p_^2^=0.37). An interaction between condition and side, (*F*(2, 54)=4.54, *p*=.015,η_p_^2^=0.14), was such that active side responding was greater for increased cost compared to FR1 (all other effects p’s>.16).

***Reward, punishment and earning.*** ANOVAs on rewards or punishment revealed no significant effects, though there was a marginal effect for condition on reward (*F*(2, 54)=2.66, *p*=.079), with greater reward rates for increased cost (M=4.27,SD=1.96) compared to FR1 (M=3.01,SD=1.57). Condition influenced earnings, (*F*(2, 54)=3.24,*p*=.047, η_p_^2^=0.11), with *post-hoc* analyses indicating this stemmed from greater earnings in increased cost (M=£3.92,SD=4.34) than FR1 (M=£0.77,SD=3.94).

***Questionnaires.*** As reported in the main analyses, there were no significant correlations.

**Experiment 3**

***Observing.*** One participant observed at baseline and none under punishment.

***MBP.*** A three-way ANOVA included observing style, condition and side. MBP were lower in punishment (M=60.17, SD=16.36) compared with baseline (M=70.15, SD=10.46), (*F*(1, 18)=13.328, *p*=.0018, η_p_^2^=0.43). MBP was greater for active (M=71.82,SD=18.67) versus inactive side (M=58.50,SD=10.28), (*F*(1, 18)=11.09, *p*=.004, η_p_^2^=0.38). Observing style was marginal (*F*(1, 18)=3.96, *p*=.06), with greater MBP in high (M=70.25, SD=6.49) versus low observers (M=60.07, SD=14.75). No other effects were significant (all p’s>0.33).

***Reward, punishment and earnings.*** Participants earned more rewards in baseline (M=4.33,SD=1.33) compared with punishment (M=2.93,SD=1.00), (*F*(1, 18)=11.17, *p*=.004, η_p_^2^=0.38) and had higher earnings (*F*(1, 18)=67.80, p<.001). Previously high observers experienced more punishment (M=2.51,SD=0.90) than low observers (M=1.66,SD=0.74) (*F*(1, 18)=5.34, *p*=.033).

**Experiment 4**

***Observing.*** Two controls and 5 patients observed infrequently, with no group difference (*p*=.21).

***MBP.*** An ANOVA with group, observing style, side and condition revealed interactions between group and condition (*F*(1, 38)=7.83, p<.01, η_p_^2^=0.17), observing style and condition (*F*(1, 38)=4.25, *p*=.046, η_p_^2^=0.10), and side and condition (*F*(1, 38)=5.64, *p*=.02, η_p_^2^=0.13). During baseline, but not punishment, controls responded more than patients and low observers responded more than high observers. An interaction between group, observing style and side (*F*(1, 38)=7.72, p<.01, η_p_^2^=0.17), stemmed from the previously low observing controls responding more on the inactive side compared to previously high observing controls with no such difference in patients.

***Reward, punishment and earnings.*** Participants earned fewer rewards in punishment (M=1.79,SD=0.72) compared with baseline (M=2.45,SD=0.83), (*F*(1, 38)=24.49, p<.001, η_p_^2^=0.39). While there were no effects on punishment, an ANOVA on earnings revealed greater baseline earnings (M=£4.86,SD=1.65) with participants losing on average in punishment (M=£-0.13, SD=1.27), (*F*(1, 38)=121.37, p<.001, η_p_^2^=0.76). There was also a group by observing style interaction (*F*(1, 38)=7.45, p<.01, η_p_^2^=0.16), due to a crossover, though no pairwise comparison reached significance.

***Questionnaires.*** The results combining data from Experiments 3 and 4 are shown in Table S2, and are consistent with the results reported in the main text.

Table S1. Correlations between task performance and individual characteristics for low and high observers in Experiment 1.

|  | Depression | Intolerance of Uncertainty | OC symptoms | State Anxiety | Trait Anxiety |
| --- | --- | --- | --- | --- | --- |
| Low observers |  |  |  |  |  |
| Observing - baseline | .16 | .16 | .34* | .42* | .48* |
| MBP-baseline | -.34* | -.12 | -.36* | -.34* | -.28 |
| High observers |  |  |  |  |  |
| Observing - baseline | .00 | .07 | -.01 | -.01 | .01 |
| MBP-baseline | -.03 | .14 | .13 | .03 | .09 |

Note, * p<.05, ** p<.01

Table S2. Correlations between task performance and individual characteristics for low and high observers in Experiments 3 and 4.

|  | Depression | Intolerance of Uncertainty | OC symptoms | State Anxiety | Trait Anxiety |
| --- | --- | --- | --- | --- | --- |
| Low observers |  |  |  |  |  |
| Observing - baseline | .20 | .36* | .33 | .22 | .27 |
| MBP-baseline | -.20 | -.34 | -.38* | -.27 | -.39* |
| High observers |  |  |  |  |  |
| Observing - baseline | .39* | .38* | .48** | .37* | .39* |
| MBP-baseline | -.43* | -.31 | -.54** | -.41* | -.38* |

Note, * p<.05, ** p<.01
